# Prizes at the Académie De Médecine

**Published:** 1859-03

**Authors:** 


PRIZES AT THE ACADÉMIE DE MÉDECINE, FOR 1858.—The Academy prize of one thousand francs, on the application of the microscope; the Capuron prize, on the death of the infant during parturition; the Civrieux prize, on the difference between neuralgia and neuritis; and the Barbier prize, for the cure of diseases generally thought incurable, have not been adjudged, on account of deficiency of memoirs, or insufficiency of merit.

M. Bauchet obtained the Portal prize, on the pathology, treatment, and diagnosis of ovarian cysts. The Itard prize, for the best work of two years standing, on practical medicine or applied therapeutics, has been adjudged to M. Duchenne. The Argenteuil prize of twelve thousand francs, on the greatest improvement in the treatment of stricture of the urethra or other diseases of the urinary organs, was not awarded to any one author, but was divided in sums varying from four thousand to one thousand francs among six competitors.

